# Care for hospitalized children undergoing institutionalization in light of the psychodynamics of work

**DOI:** 10.1590/0034-7167-2024-0158

**Published:** 2025-03-31

**Authors:** Daniela Giotti da Silva, Maria de Lourdes Custódio Duarte, Eduarda Paza Dias, Alessandra Porto d’Ávila, Karolaine Aparecida Borba Lopes

**Affiliations:** I Universidade Federal do Rio Grande do Sul. Porto Alegre, Rio Grande do Sul, Brazil

**Keywords:** Child, Hospitalized, Nurses, Pediatric, Child, Foster, Work, Institutionalization., Niño Hospitalizado, Enfermeras Pediátricas, Niño Acogido, Trabajo, Institucionalización.

## Abstract

**Objectives:**

to analyze the factors of pleasure and distress of nurses in the care of hospitalized children undergoing institutionalization in a pediatric unit.

**Methods:**

a qualitative descriptive study, carried out in a university hospital in southern Brazil, with the participation of 11 nurses. Information was collected through semi-structured interviews and analyzed according to thematic analysis proposed by Minayo, resulting in two analytical categories.

**Results:**

nurses reported professional fulfillment, involvement in health education, and bonding with children as factors that generated pleasure at work. However, cases of child abuse, disengagement at discharge, and invasive procedures were mentioned as factors of distress.

**Final Considerations:**

carrying out this study provided an opportunity to understand the specificities of feelings of pleasure and distress verbalized by nurses in light of the Psychodynamics of Work, helping managers to articulate practices that provide reflection on the topic in work environments.

## RESUMEN

**Objetivos:**

analizar los factores de placer y sufrimiento de enfermeras en el cuidado de niños hospitalizados en proceso de institucionalización en una unidad pediátrica.

**Métodos:**

estudio descriptivo cualitativo, realizado en un hospital universitario del Sur del Brasil, con la participación de 11 enfermeras. La información fue recolectada a través de entrevistas semiestructuradas y analizada según el análisis temático, propuesto por Minayo, emergiendo dos categorías analíticas.

**Resultados:**

las enfermeras refirieron la realización profesional, el trabajo en educación para la salud y el vínculo con los niños como factores que generan placer en el trabajo. Sin embargo, se mencionaron como factores de sufrimiento casos de violencia infantil, desvinculación en el momento del alta y procedimientos invasivos.

**Consideraciones Finales:**

la realización de este estudio brindó la oportunidad de comprender las especificidades de los sentimientos de placer y sufrimiento verbalizados por las enfermeras a la luz de la Psicodinámica del Trabajo, ayudando a los gestores a articular prácticas que propicien la reflexión sobre el tema en el ambiente de trabajo.

## INTRODUCTION

Institutional care, a term used by the Statute of Children and Adolescents (In Portuguese, *Estatuto da Criança e do Adolescente* - ECA) to designate institutionalization, is a protective measure provided for in Law 8,069/90, which must be applied whenever children’s and adolescents’ rights are threatened or disrespected. Thus, the ECA provides for care in cases of distress in any form of negligence, discrimination, exploitation, violence, cruelty and oppression, whether due to omission by society, the State and/or parents or guardians^(1)^.

In this context, based on the ECA assumptions, children are recognized as an absolute priority, since they are in a position to develop and have legally protected rights^(1)^. This care does not imply deprivation of liberty and offers personalized care, prioritizing decentralized and municipalized actions^(2)^.

Although institutionalization is a time that involves removing children from their conventional environment, many of them have comorbidities that require hospitalization, making this process even more complex. In this context, these children tend to become more fragile and vulnerable, especially when they do not have a significant other to support their feelings, such as a mother, father or someone else with a type of bond that is not necessarily blood-related^(3)^.

In this logic, the nurses who provide assistance to them seek to develop and satisfy their care demands and, as far as possible, include emotional aspects in the therapeutic relationship^(4)^.

In this regard, affection is strongly present in the attitudes, actions and environments of nursing care in a pediatric context^(4)^. In other words, in addition to being holders of theoretical and practical skills, they demonstrate affective involvement in the interpersonal relationships they establish, with a view to contributing to hospitalized children’s health and well-being, in the search for comprehensive care^(5)^.

Nursing students and nurses show that the relationship of care, closeness and emotional involvement alleviate stress, functioning as a compensatory mechanism, as they experience not only distress, but also gratifying moments in the relational process, such as the promotion of healing. Therefore, negative emotions and emotional exhaustion resulting from nursing practice are mitigated by positive emotions such as gratification and satisfaction^(6)^.

In this logic, the Psychodynamics of Work (POW) privileges the clinic as a way of constructing knowledge, interpreting and analyzing work environments, positioning itself as an instrument capable of understanding the health-disease process^(7)^. For POW, work is understood as an ontological category with the potential to be a source of pleasure or distress^(8)^.

Pleasure can manifest itself as a beneficial feeling related to workers’ psychological stability, from the moment they overcome the difficulties imposed by work, through acquired skills and problem-solving actions in their professional environment^(8-13)^. However, distress is considered an unpleasant emotional experience associated with feelings such as worthlessness, impotence, dissatisfaction and stress, in the face of work conditions and organization that, linked to people characteristics, can lead to psychological illness^(9)^.

In the work context of the pediatric inpatient unit, the bond between nurses and children undergoing institutionalization is often overlapped and intertwined as an “almost parental” relationship^(14)^. Thus, it is common for this relationship between worker and child to generate satisfaction, but also distress, due to increased psychological burden arising from the emotional bond with these children who are hospitalized for a long period of time without companions.

Studies were found on the following topics: children in foster care institutions from the perspective of the multidisciplinary team^(2)^; hospitalized children dependent on technology from the perspective of family members and the nursing team^(3)^; feelings of nurses in caring for children hospitalized without a companion in an integrative review study^(4)^; the role of nursing in foster care institutions for children from the perspective of the multidisciplinary team^(5)^. Moreover, studies on POW involving pleasure and distress in pediatric oncology units^(15)^, pediatric therapy units^(16)^ and pediatric emergency services^(17)^ were common in the searches, as these are fields widely explored in the literature. However, studies that address the topic involving nurses’ care for hospitalized children who are in the process of institutionalization using the POW framework were not found in the literature, justifying the relevance of this study.

In view of this, it is important to conduct research that investigates, from the perspective of POW, the impact that the care offered by the nursing team to children in the process of institutionalization has on the lives of these workers. Based on these considerations, the present study will seek to answer the following guiding question: what are the factors of pleasure and distress for nurses in caring for hospitalized children in the process of institutionalization in a pediatric unit?

The results of this study are intended to help in understanding the specificities of the feelings of pleasure and distress of these workers in light of POW. The study can also contribute to discussing issues related to workers’ health in the studied site, as it gives visibility to distress of this category, contributing to triggering a process of reflection on the topic.

## OBJECTIVES

To analyze the factors of pleasure and distress of nurses in the care of hospitalized children undergoing institutionalization in a pediatric unit.

## METHODS

### Ethical aspects

The study was approved by the health institution’s Research Ethics Committee (REC), respecting the items contained in Resolution 466/12^(18)^ and Resolution 510/2016^(19)^ of the Brazilian National Health Council (In Portuguese, *Conselho Nacional de Saúde* - CNS). Participants were assured of the preservation of their identities and were provided with the Informed Consent Form (ICF), with copies of the same content for interviewees and the researcher.

### Study design

This is a qualitative descriptive study. Qualitative research is concerned with what cannot be quantified, i.e., it works with a universe of meanings, which corresponds to relationships, processes and phenomena that cannot be reduced to the operationalization of variables. It aims to explore the meanings of human actions and relationships, a side that is not perceptible and captured in statistics^(20)^.

The research used the COnsolidated criteria for REporting Qualitative studies (COREQ) for developing and writing the manuscript. Thus, it strictly met the 32 criteria of the three domains: research team and reflexivity, study concept, analysis and results that allowed qualifying the study^(21)^.

### Study setting

The study was conducted at a university hospital in southern Brazil, in the pediatric inpatient unit belonging to the Pediatric Nursing Service. The unit has 28 beds, consisting of six wards with four beds in each, totaling 24 beds exclusively for the Brazilian Healthcare system (In Portuguese, *Sistema Único de Saúde* - SUS) and two rooms with two semi-private beds, intended for health insurance companies. This unit receives hospitalizations of children with chronic diseases and comorbidities, including genetic diseases, acute clinical care, preand post-surgical care.

Since it is considered a semi-critical unit^(22)^, the institution recommends that a professional from the health team, especially a nurse, remain full-time in each of the wards. In this way, the unit has become a reference for children with a history of social vulnerability who, in some cases, are unaccompanied, and this presence of professionals naturally creates a closer relationship between hospitalized children and these workers. This reason influenced the choice of location.

### Study participants

The unit has 13 nurses, and all were invited to participate in the study. Nurses who were performing care functions for hospitalized children undergoing institutionalization and who had an effective contract of six months or more to provide greater familiarity with cases of interest to the study were included. Nurses who were on sick leave, pregnancy leave or vacation were excluded.

The interviewees were chosen intentionally by invitation, involving the selection of participants who share particular characteristics and have the potential to provide rich, relevant and pertinent data to the research question^(20)^. It is worth noting that it was decided to interview the unit’s nurses due to the fact that these professionals taking a leading role in care, since they live with children at the bedside full time.

After applying the inclusion and exclusion criteria, one nurse was on sick leave due to flu-like symptoms, and another nurse had been recently hired and did not have at least six months of experience. Coincidentally, both nurses excluded due to the selection criteria were male. Therefore, 11 nurses met the selection criteria and participated in the study.

### Data collection and organization

The interviews took place after the research project was presented to all nurses in the unit at a team meeting. Individual interviews were then scheduled with professionals interested in participating in the study outside of their work hours.

Data collection was carried out from April to May 2023, with interviews lasting between 18 and 26 minutes, and were administered by one of the researchers with the assistance of a scientific initiation scholarship holder, both with previous experience in qualitative research collection. The interviews took place in person, in a meeting room of the unit, without noise and with privacy guaranteed, and were recorded and transcribed in full for better analysis and reading of the material.

The data collection instrument was a semi-structured interview containing closed-ended questions about the profile of professionals and open-ended questions about the feelings and factors of pleasure and distress aroused when caring for a hospitalized child undergoing institutionalization in the pediatric unit.

The professionals interviewed were coded by the letter “W”, for worker, followed by the number according to the sequence in which the interviews took place (for instance, W1, W2, and so on).

### Data analysis

The data were analyzed according to the thematic content analysis proposed by Minayo^(20)^, and the analysis process was divided into three stages: pre-analysis; material exploration; and treatment of results obtained and interpretation.

The first stage consisted of reviewing the initial hypotheses and objectives of the research. During this phase, a cursory reading was carried out to ensure direct and intense contact with the field material, in order to understand the *corpus* content and constitution. Also in the pre-analytical phase, the recording units (keyword), the context unit (the delimitation of the context for understanding the recording unit), the excerpts, the form of categorization, the coding modality and the more general theoretical concepts that guided the analysis were determined^(20)^.

The second stage consisted of grouping categories where content was organized, aiming to achieve understanding of the text, separating it into categories responsible for specifying the topics^(20)^.

Finally, in the third stage, the information obtained was highlighted and, from this, inferences and interpretations were made, relating them around new theoretical dimensions based on literature^(20)^.

After thematic content analysis, two analytical categories emerged: Factors that generate pleasure at work when caring for hospitalized children undergoing institutionalization; and Factors that generate distress at work when caring for hospitalized children undergoing institutionalization.

The analyses and discussions of results were carried out in light of POW, serving as a theoretical framework for the study. POW was conceived by French psychiatrist Christophe Dejours, who studies the dynamic relationships between work and the subjective and intersubjective processes that arise from the relationship between the psychic organization of people and the work process^(8-13)^.

## RESULTS

Participants’ age ranged from 26 to 46 years, and all participants were female. Regarding the time since training of workers, one nurse had three years of training; one had four years; three had 13 years; two had 18 years; three had 20 years; and one had 21 years, the nurse had the longest training time in the study unit during the data collection period. As for job tenure in the health institution’s pediatric unit, one nurse had a six-month employment contract (which was one of the requirements for inclusion in the study), and the professional with the longest contract time had 16 years in the unit studied.

### Factors that generate pleasure at work when caring for hospitalized children undergoing institutionalization

Considering the above and based on nurses’ statements in the pediatric inpatient unit studied, the following subcategories emerged: professional achievement; performance in health education; and affection and bond with the child.

Professional fulfillment occurs for several reasons, according to the workers interviewed. One of the factors that generates pleasure and contentment is the feeling of duty fulfilled and leaving work environments with the feeling that they made a difference in the care of hospitalized children.

*These are children who need us the most. It gives me satisfaction to know that the care for that child is very important* [...]. *So, when I go to do the procedure, I take the opportunity to talk and even hold them, if possible. This is important for children; it gives me satisfaction.* (W5)

*When they are admitted, they are very weak, with a fever, and their ventilatory problems are getting worse. And as the days go by, we continue to provide care, we start to see results and we are happy, because this child arrived in a poor state of health and is getting better. We feel happy, because they were restless children, they would only cry and sleep and soon they are interacting with you* [...]. *It is very gratifying; it is a feeling of joy.* (W4)

The interviewees reported pleasure in demonstrating the importance that work has in their lives and in verbalizing how much they enjoy what they do. They feel professionally fulfilled when they identify with their work, and derive pleasure from making a difference in children’s hospitalization process, especially when there is an improvement in children’s health status.

According to interviewees, seeing children’s clinical progress and progress, who were previously very weak, is an aspect that generates pleasure and satisfaction, since the favorable results were due to their participation and investment in care. Thus, it is possible to see that active participation in patient improvement is a condition that generates pleasure and satisfaction, since the outcomes, most of the time, with positive and favorable impacts, are motivating for continuation of work.

Work and training in health education were highlighted as one of the competencies of the workers interviewed in preparing these children for hospital discharge. When carrying out this work, they report that they feel comfortable, as they are referring children to caregivers who will be trained to do so at the institution where children will be received.

*One thing that comforts me and makes me very happy is when I get to meet the people from the institution, when they come beforehand. We can even do some training or something before children leave, I think that gives a feeling of comfort* [...]. *When the nurse from the institution or social worker and physiotherapist come, we can do “shift handover”. This is very important to me. I feel good when I can do it. It feels like your work is complete. You gave back to society, or to the institution, but you handed over the patient. This is interesting, because otherwise I think it seems a bit incomplete or dubious.* (W2)

Nurses recognize the importance of interpersonal relationships in health practices to understand children’s health/illness situation (often dependent on health technologies) and to provide care training. Thus, planning the hospital discharge process and equipping future caregivers at the institution to which children are transferred provides the feeling of completing a job well, generating pleasure in work.

Affection and bonding with children were also highlighted by interviewees as factors that generate pleasure as they give themselves to that child who lives for long periods in hospital and unaccompanied. Nurses verbalized that attitudes such as holding children in their arms and talking while looking them in the eye are details that bring them closer and make a difference in care.

*Because they lack extra affection, family care. So, we usually have to do this role of motherhood in addition to nursing care. We end up becoming the children’s reference caregiver. So, we end up becoming much more attached* [...]. *We end up trying to fill in some missing issue in this child’s life.* (W6)

*I feel much more obligated to offer as much love, affection and wholeness as possible. Our routine is very busy, but I manage to reconcile it in a way that brings me great pleasure. I really enjoy being able to give of myself to these special children* [...]*. I think that whatever we can provide is valid, it makes a difference. The affection that that child doesn’t have, holding them in my arms, looking them in the eye. These little details bring me pleasure, because I know that, when I can minimize a child’s distress, it is something that does me a lot of good.* (W9)

It is almost inevitable to work in a pediatric unit and not create a bond with a hospitalized child. This situation is even more so when children are unaccompanied and in need of special attention. Thus, the relationship formed by affection, affinity, interaction, closeness and bonding generates a feeling of pleasure for professionals who find a moment in the midst of their busy routine to perform mothering. Furthermore, “mothering” is nothing more than a job that is not contractually prescribed, but which becomes a reality in the routines of pediatric workers, generating pleasure.

### Factors that generate distress at work when caring for hospitalized children undergoing institutionalization

From the interviews with nurses in the study unit, the following subcategories emerged: cases of violence; disengagement and detachment; and invasive procedures.

The nurses interviewed stated that working with cases involving child abuse is very difficult and painful, as feelings of pity and anger often arise, in addition to insecurity related to the lack of knowledge about caring for these children in this context.

*It awakens different feelings, when a child goes through some type of abuse, whether psychological or sexual, we feel angry, because this is a person who depends on an adult and is distress aggression. It is a feeling of anger* [...]. *Children end up saying some things that you don’t expect during play. We are not trained to know who we should call to try to solve these problems* [...]. *I think we should be equipped to know what to do when these situations happen, because it is not covered much during our training as nurses.* (W4)

*A child who suffers violence is something that really affects me. It’s something I can’t understand. Many times, they arrive with “suspicion” and the parents are there* [...]. *There is a feeling of repulsion, just knowing what may have happened to the child, but I don’t judge, I don’t say anything to them, but I feel it. It causes a blockage.* (W11)

It is clear, through workers’ statements, that the care and assistance provided to cases of violence against children in the midst of the daily work routine are tiring and exhausting. The high emotional and psychological burden involved is likely to cause distress, capable of inducing a process of illness caused by work environments. Nurses also reinforced in the interviews that the lack of preparation and experience in treating these cases ends up interfering with the way they provide care, since the lack of knowledge of the intraand extra-hospital network generates negative feelings and insecurity among workers.

The interviewees highlighted that the experiences they have at work often cause helplessness, which comes from situations in which professionals, despite providing qualified assistance, are unable to change patients’ condition. Furthermore, carrying out the reverse process of disengagement and detachment at the time of institutionalization is a source of distress for professionals who have cared for children for a long period.

*It’s a difficult detachment* [...]. *Some institutionalizations are quicker, and others take longer, and for these children who are left unattended for a long time, we end up replacing the family at various times. Separation is painful.* (W2)

*Sadness due to separation, fear. We feel the fact that the child is leaving, because the nursing staff, until then, was taking care of this child, and now the child is leaving here. So, I think sadness is a factor of distress in the process of separation, institutionalization.* (W6)

Nurses suffer when they have to repress their feelings and work internally through the process of hospital discharge and the breaking of the bond developed throughout the care of children, at the time children are transferred to the foster care institution. Moreover, they feel insecure due to the lack of knowledge about children’s future, increasing the psychological burden at work.

Invasive procedures represent one of the main negative experiences in hospital settings, and cause anxiety and fear in children, generating great distress for the workers who have to perform them.

*When a child has been hospitalized for a long time and has been handled a lot, we ask ourselves, “Did I really need to collect this urine by catheterization?”. You start to think that maybe it could bring more distress to that child instead of relief, that life outside was so difficult; in some cases, I think that this gets in the way. We automate these processes so that the child doesn’t suffer so much, but these aren’t good things.* (W2)

*We wonder how the child is doing, if they are okay. You performed a procedure, you leave the shift that day, and you see that the child is crying more, with pain in their face, and you get worried* [...]. (W4)

The challenge for nurses to care for a child through painful procedures causes even more distress for the workers interviewed, given that they know that children are already under stress due to previous issues beyond hospitalization. Therefore, according to professionals’ reports, automation of procedures is used as a way to reduce their own distress.

## DISCUSSION

The pleasure derived from the professional achievement of interviewees occurs when they are able to monitor improvement in children’s health. Moreover, exchange of information with the team at the institution that will receive the children was also highlighted as an aspect of pleasure, as workers are able to carry out transition of care through health education of future caregivers at the time of children’s discharge. In relation to prolonged time spent in hospital settings, these professionals also create a bond with these children, since they highlight the lack of a companion, causing them to spend more time offering affection, love and comprehensive care.

Nurses’ job satisfaction is conditioned by a multitude of factors: feeling good about what they do, being happy and fulfilled at work, doing what they like, having their needs met and having their expectations at work met. Thus, satisfaction occurs when their professional desires meet the institution’s goals and philosophy, when they achieve an expected result or when the desired outcome is not discarded^(23)^.

Recognition of the quality of work performed is the response to the subjective expectations that professionals carry. Thus, challenges and fatigue can be alleviated by the feeling of pleasure with recognition in work environments. In this way, working is not only about producing, but also about transforming oneself^(13)^, in the pursuit of a common goal; in this case, improving children’s health.

Feelings of satisfaction and gratification occur when professionals understand the restoration of patients’ health as a consequence of nursing interventions during the hospitalization period. It is considered that care does not occur in isolation, being an interactive process, requiring availability, trust and receptiveness by workers^(15)^.

When the time comes for institutionalization, children need to adapt to a new reality, and caregivers play a fundamental role in understanding the particularities and potential of each child. In addition to this, the host institution has the role of providing care and education, providing resources to cope with difficulties and favoring the emotional, cognitive and social development of these children^(24)^.

Thus, to establish a coordinated network, communicative action is essential, implying interpersonal relationships of interdependence, which guarantee access and continuity of care and, mainly, avoid unnecessary procedures or their duplication^(25)^.

Contributing to this, it is essential that nursing professionals provide training for those who will receive children dependent on healthcare from a hospital service, with specific technical knowledge that can support the caregivers who will provide this care^(24)^.

Thus, satisfaction in performing a prescribed task, including real work actions, contributes to the quality of interpersonal relationships, based on organization of work, and has an impact on positive experiences for individuals in their professional practice^(8)^. Thus, pleasure is extracted from the success of work, and may be the result of good relationships in work contexts, i.e., the recognition of one’s work in carrying out health education^(17)^.

When this experience is added to the fact that children have a fragile support network or even no companion during this period, it becomes an even greater challenge for workers who care for them. In this regard, healthcare professionals, by establishing a bond with children, can outline possibilities that allow the maintenance of children’s development in all its aspects. Thus, nurses establish an interpersonal relationship with future caregivers, as well as with hospitalized children, who in themselves already experience situations that cause stress and emotional destabilization^(26)^.

Thus, bonding and affection, although not prescribed in nursing professionals’ work, occur in the daily work, since nurses spend most of their shift at the bedside with children. Furthermore, creativity is essential to transform prescribed work into real work, because without this ability, work environments can become limiting and tiring^(12)^.

Establishing a bond and emotional involvement makes nurses’ daily work more effective, as it allows for greater trust in the relationship between children and professionals. The bond itself is a two-way street, as it brings people closer together and improves quality of care, and also enhances the human resources used by workers, going beyond general protocols and leading to greater commitment to care^(23)^.

Considering the fact that all interviewees were female, those who were also mothers highlighted common aspects in their statements, i.e., a brief association between the care provided in the hospital sector and motherhood, both in the way of acting and in the emotional involvement with children. Historically, the bond created between pediatric professionals and children is more intense when compared to other areas, making these workers remember the children who are at home. Hence, the care provided to children requires these nurses to develop interactive skills that directly contribute to the bond and the achievement of trust, and this can imply an intensity of the feelings generated, due to prolonged time of care^(27)^.

Thus, subjective mobilization is the process through which workers engage in work, use their subjectivity, practical intelligence and the work collective to transform situations that cause distress (the lack of affection and children’s family of origin) into situations that generate pleasure (bond and reciprocity of affection between worker and child)^(7)^. Therefore, situations that cause distress to workers occur when there is a failure in the mediation between workers’ expectations and the reality imposed by work organization and management^(16)^.

Subjective identification with work presupposes that the activity has a rewarding meaning for the subject in the form of symbolic retribution who, by using their personality and intelligence, confronts the subjective reality of work and finds ways to transform their distress into something positive, which is called creative distress. Pathogenic distress, on the other hand, develops from the moment in which workers do not have the freedom to act and transform their work reality, using defensive strategies to adapt to work, which are alienating to workers^(8)^.

Distress was evidenced in this study based on workers’ experiences when dealing with cases of violence committed, in a variety of ways, against children. Furthermore, because they have a strong bond with children, negative feelings arise when this connection is broken, at the time of separation for institutionalization, generating a forced detachment and a feeling of belonging. Furthermore, when performing invasive procedures, these professionals are shaken by generating more discomfort for this already fragile child, often automating this process to alleviate their psychological burden.

Contact with violence against children awakens feelings of distress in nurses, as they seek to understand the situation in which it occurs. Thus, seeking to understand this context is a complex task for professionals, since the family should be the foundation, protecting, welcoming and providing security and comfort throughout their growth and development^(28)^.

In this scenario, when the aggressor is a family member, the approach to the child victim of violence who is hospitalized is difficult to manage, since these practices are perpetuated by different beliefs and generations. The attempt to remain impartial, without judging the family and continuing to care for children, generates distress and emotional exhaustion in workers, causing an increase in the psychological burden and making work tiring^(11)^.

In this way, dealing with issues of violence in childhood has a great impact on work environments for workers, since the lack of autonomy and management is an element that negatively influences the relationship between nurse and family member, as there are still barriers to training and qualification in the face of situations of violence against children and adolescents^(17)^.

In general, healthcare professionals have difficulty working with situations involving violence, regardless of their level of training, and this is due to the fact that health training assumes a biomedical view, which operates in a curative logic, not considering the complexity of the issue, which is not susceptible to medicalization, requiring other strategies from nurses. Thus, the focus of professional interventions in decision-making regarding institutional care for children may be linked to the difficulty of handling situations involving greater complexity^(29)^, since, often, children’s “support network” was the same one that practiced violence against them.

Thus, workers are subject to emotional exhaustion, given the experiences that result from the multi-purpose nature of care, as workers bond with children throughout the hospitalization period^(30)^. Thus, the role played by workers with these children is compared to that of their own children, with the same concerns and dedication. However, the emotional bond with children who will be taken in results in the breaking of the bond at the time of discharge from hospital on the way to the care institution. This process causes psychological exhaustion, increasing feelings of abandonment and demotivation for the professionals who need to be used to the absence of children^(24)^.

These feelings can be seen as synonymous with professional limitations and impotence in the workplace. Professionals feel frustrated and, in addition to dealing with the experience of separation from children, they also deal with the unexpected and tragic feeling of not belonging to the situation that was part of their work routine^(16)^.

This feeling of helplessness goes beyond hospital settings, as nurses would like to be able to contribute to these children beyond this scope, believing that, in order for children to experience a better life, support from other support networks would be necessary. Thus, workers would like to have autonomy to be able to continue providing care for children, but their role as a professional implies certain attitudes that remain below their desires^(28)^.

Although care related to the body and illness aims at the recovery and quality of life of hospitalized children, nurses, in their care practice, can perform invasive procedures that are necessary and are part of treatment; however, they generate discomfort in children and, consequently, can cause distress to workers^(31)^.

Children with complex chronic conditions end up needing to be punctured (venous and arterial punctures, blood glucose checks), aspirated, changed position, subjected to the insertion of probes, dressings, catheterizations, among others. Thus, the interface between technique and intersubjectivity is strategic in this complex environment, in which care sometimes involves pain, invasion and containment^(32)^, and can trigger stress and anxiety reactions in both children and nurses, who need to know how to manage the situation^(33)^. Therefore, impositions of technical and scientific mastery that are placed on these professionals’ work experience, at a time when children are very fragile, can cause workers to become mentally ill.

Furthermore, even though nurses have considerable experience in the area, they continue to empathize with the distress of these children as if they were their own family members^(15)^. Thus, workers need to deal with reality and the difficulties imposed, recognizing the free exercise of their intelligence and problem-solving skills^(10)^. It is at this moment that distress affects professionals, as the bond created triggers the feeling of causing pain in children.

Therefore, going to the bedside to perform only the procedure does not seem to be an option in pediatrics (prescribed work). It is necessary to have a relationship of trust, bond, and proximity when trying to convince children and assist in the resilience process (real work). Thus, it is in the distance between expected norms and rules and the way of working in reality that the possibility of distress occurs^(11)^, since the care of these children is not just about technical work.

### Study limitations

It is important to emphasize that this study has some limitations, such as the choice of only one specific pediatric inpatient unit of a single hospital, since it is possible that, in other units that receive children with the profile of the study, workers may have different perceptions of factors of pleasure and distress. Furthermore, the particularity of this study in interviewing nurses presented a cutout of only one professional category. Therefore, the inclusion of other professionals from the multidisciplinary team could certainly add more information about POW in these units.

### Contributions to nursing, health or public policy

The study contributed to the discussion on issues related to workers’ health in the site studied, as it gives visibility to the distress of this category. In addition to this, it promotes the attempt to assist and provide the institution’s managers with subsidies and tools for articulation and implementation of practices that provide reflection on the topic in the workplace, according to the needs of workers who find themselves in settings that trigger illness due to the high psychological burden.

## FINAL CONSIDERATIONS

Nurses found pleasure in their professional achievement, their work in health education, and their affection and bond with children. These aspects reverberate feelings of pleasure in workers when they realize that their work to improve children’s health status was the result of continuous work, consisting of bonding and closeness with children, generating professional satisfaction. Moreover, the feeling of a job well done and mission accomplished occurs when workers train children’s future caregivers at the institution.

Cases of violence, detachment and invasive procedures were reported by workers as aspects that generate distress in their daily work routine. Professionals’ distress when they work in cases of violence is explicit, and the process of detaching from children when they are institutionalized is a very difficult task. Furthermore, performing invasive procedures on a child who has already suffered outside the hospital walls generates an increased psychological burden.

Considering care in hospital care, it is important to encourage new studies that promote the involvement of care for children in the process of institutionalization in other professionals in the multidisciplinary team, ensuring an even more accurate understanding of the repercussions of these feelings on health and work processes.

Therefore, this study provided an opportunity to understand the specificities of feelings of pleasure and distress expressed by nurses when caring for hospitalized children who are in the process of institutionalization in a pediatric unit of a general hospital in light of POW.

## References

[1] Presidência da República (BR) (1990). Lei nº 8.069, de 13 de julho de 1990. Dispõe sobre o Estatuto da Criança e do Adolescente e dá outras providências.

[2] Fermino S, Lima DB. (2023). Análisis de las condiciones psíquicas de niños y adolescentes en institucionalización. Rev Psicol, Divers Saúde.

[3] Depianti JR, Cabral IE. (2023). Hospitalized children with complex special healthcare needs: multiple case studies. Acta Paul Enferm.

[4] Diogo P, Baltar P., Sequeira C, Carvalho JC, Sá L (2014). Determinantes afetivos de cuidar da criança hospitalizada sem acompanhante: o trabalho emocional em enfermagem. IV Congresso Internacional ASPESM. Padrões de Qualidade em Saúde Mental.

[5] Vasconcelos J, Troncoso MP, Backes DS. (2021). Atribuições da enfermeira em um serviço de acolhimento institucional para crianças e adolescentes. Cienc Enferm.

[6] Smith P. (2017). The Emotional Labour of Nursing Revisited: can nurses Still Care? 2ª Ed. Red Globe Press.

[7] Mendes AM. (2007). Psicodinâmica do trabalho: teoria, método e pesquisas.

[8] Dejours C. (2015). A loucura do trabalho estudo de psicopatologia do trabalho.

[9] Marks J. (2019). The psychodynamic analysis of work. Modern & Contemporary France;.

[10] Dejours C. (2007). Psicodinâmica do Trabalho: contribuições da Escola Dejouriana à análise da relação prazer, sofrimento e trabalho.

[11] Dejours C. (2012). Psicodinâmica do trabalho e teoria da sedução. Psicol Estud.

[12] Dejours C. (2012). Trabalho Vivo: sexualidade e trabalho.

[13] Dejours C. (2017). Psicodinâmica do Trabalho: casos clínicos.

[14] Lulgjuraj D, Maneval RE. (2021). Unaccompanied Hospitalized Children: an integrative review. J Pediatr Nurs.

[15] Duarte MLC, Glanzner CH, Bagatini MMC, Silva DG, Mattos LG. (2021). Pleasure and suffering in the work of nurses at the oncopediatric hospital unit: qualitative research. Rev Bras Enferm.

[16] Vasconcelos LS, Camponogara S, Dias GL, Bonfada MS, Beck CLC, Rodrigues IL. (2019). Prazer e sofrimento no trabalho de enfermagem em unidade de terapia intensiva pediátrica. Rev Min Enferm.

[17] Lamb FA, Beck CLC, Coelho APF, Vasconcelos RO. (2019). Nursing work in a pediatric emergency service: between pleasure and pain. Cogitare Enferm.

[18] Ministério da Saúde (BR) (2013). Resolução nº 466, de 12 de dezembro de 2012. Dispõe sobre diretrizes e normas regulamentadoras de pesquisas envolvendo seres humanos.

[19] Ministério da Saúde (BR) (2016). Saúde Legis.

[20] Minayo MCS. (2014). O desafio do conhecimento: pesquisa qualitativa em saúde.

[21] Tong A, Sainsbury P, Craig J. (2007). Consolidated criteria for reporting qualitative research (COREQ): a 32-item checklist for interviews and focus groups. Int J Qual Health Care.

[22] Ministério da Saúde (BR) (2022). Dispõe sobre produtos com ação antimicrobiana utilizados em artigos críticos e semicríticos, e seu registro.

[23] Almeida DMG. (2020). Satisfação profissional e engagement: perceção dos enfermeiros.

[24] Gabatz RIB, Schwartz E, Milbrath VM. (2019). Experiências de cuidado da criança institucionalizada: o lado oculto do trabalho. Rev Gaúcha Enferm.

[25] Sleppy RM, Watson BD, Donohue PK, Seltzer RR. (2023). Caring for Hospitalized Children in Foster Care: provider training, preparedness, and practice. Hospital Pediatrics.

[26] Rodrigues LS, Rodrigues IS, Rabelo LMT, Oliveira LB, Carmo YC, Andrade HS. (2022). Atuação da enfermagem com cuidados paliativos em crianças oncológicas. Rev Inova Saúde.

[27] Lopes ADS, Paixão TVA, Dantas HLL, Monteiro FS, Lúcio IML. (2023). Experiências da equipe de enfermagem frente ao processo de finitude de pacientes crônicos pediátricos. Gepnews.

[28] Silva MS, Milbrath VM, Santos BA, Bazzan JS, Gabatz RIB, Freitag VL. (2021). Nursing care for child/adolescent victims of violence: integrative review. Rev.

[29] Felix E, Silva AL. (2019). Acolhimento institucional de crianças e adolescentes: a compreensão da equipe multidisciplinar de saúde de um hospital de alta complexidade. Socied Deb.

[30] Waterworth S, Grace AM. (2021). Resilience and Burnout in Pediatric Nurses in a Tertiary Children’s Hospital. MCN, Am J Mat Child Nurs.

[31] Simonato MP, Mitre RMA, Galheigo SM. (2019). O cotidiano hospitalar de crianças com hospitalizações prolongadas: entre tramas dos cuidados com o corpo e as mediações possíveis. Interface (Botucatu).

[32] Santos RA. (2022). Crianças que não veem o sol.

[33] Furtado KR, Dias TL, Marchett A, Nunes EPS. (2020). O uso do jogo digital “Hospital Mirim” como estratégia de enfrentamento a procedimento invasivo. Estud Pesqui Psicol.

