# Supplementary material for: Care for hospitalized children undergoing institutionalization in light of the psychodynamics of work
Source: Rev Bras Enferm. 2025 Mar 31;78(2):e20240158. doi: 10.1590/0034-7167-2024-0158 (PMC11961043; doi:10.1590/0034-7167-2024-0158)
Supplement: Translation

# Translation: Cuidado à criança hospitalizada em processo de institucionalização à luz da psicodinâmica do trabalho

**DOI:** 10.1590/0034-7167-2024-0158pt

**Authors:** Daniela Giotti da Silva, Maria de Lourdes Custódio Duarte, Eduarda Paza Dias, Alessandra Porto d’Ávila, Karolaine Aparecida Borba Lopes

**Affiliations:** I Universidade Federal do Rio Grande do Sul. Porto Alegre, Rio Grande do Sul, Brasil

**Keywords:** Criança Hospitalizada, Enfermeiros Pediátricos, Criança Acolhida, Trabalho, Institucionalização.

## Abstract

**Objetivos:**

analisar os fatores de prazer e sofrimento do enfermeiro no cuidado à criança hospitalizada em processo de institucionalização em uma unidade pediátrica.

**Métodos:**

estudo qualitativo do tipo descritivo, realizado em hospital universitário do Sul do Brasil, com participação de 11 enfermeiras. As informações foram coletadas através de entrevista semiestruturada e analisadas de acordo com a análise temática, proposta por Minayo, emergindo duas categorias analíticas.

**Resultados:**

as enfermeiras referiram realização profissional, atuação na educação em saúde e vínculo com a criança como fatores geradores de prazer no trabalho. No entanto, casos de violência infantil, desvinculação no momento da alta e procedimentos invasivos foram mencionados como fatores de sofrimento.

**Considerações Finais:**

a realização deste estudo oportunizou conhecer as especificidades diante dos sentimentos de prazer e sofrimento verbalizados pelas enfermeiras à luz da Psicodinâmica do Trabalho, auxiliando gestores na articulação de práticas que proporcionem reflexão sobre o tema no ambiente laboral.

## INTRODUÇÃO

O acolhimento institucional, nomenclatura utilizada pelo Estatuto da Criança e do Adolescente (ECA) para designar institucionalização, é uma medida de proteção prevista na Lei n^o^ 8.069/90, que deve ser aplicada sempre que os direitos das crianças e adolescentes forem ameaçados ou desrespeitados. Dessa maneira, o ECA prevê o acolhimento nos casos de sofrimento em qualquer forma de negligência, discriminação, exploração, violência, crueldade e opressão, seja por omissão da sociedade, do Estado e/ou dos pais ou responsáveis^(1)^.

Nesse contexto, a partir dos pressupostos do ECA, a criança é reconhecida como prioridade absoluta, uma vez que se encontra em condições de desenvolvimento e com direitos juridicamente protegidos^(1)^. Esse acolhimento não implica privação de liberdade e oferece atendimento particularizado, priorizando ações descentralizadas e municipalizadas^(2)^.

Apesar de a institucionalização ser um momento que implica que a criança seja retirada do seu ambiente convencional, muitas delas possuem comorbidades que necessitam de hospitalização, complexificando ainda mais esse processo. Nesse contexto, essas crianças tendem a ficar mais fragilizadas e vulneráveis, principalmente quando não possuem acompanhamento de uma pessoa afetivamente significativa para acolher seus sentimentos, como mãe, pai ou algum outra pessoa com um tipo de vínculo não necessariamente consanguíneo^(3)^.

Nessa lógica, os enfermeiros que prestam assistência a elas procuram desenvolver e satisfazer as suas demandas de cuidados e, na medida do possível, englobar aspectos emocionais na relação terapêutica^(4)^.

Nesse sentido, o afeto está fortemente presente nas atitudes, nas ações e nos ambientes de cuidado de enfermagem em contexto pediátrico^(4)^. Ou seja, além de serem detentores de competências teórico-práticas, evidenciam o envolvimento afetivo nas relações interpessoais que estabelecem, com vistas a contribuir para a saúde e bem-estar da criança em hospitalização, na busca pela integralidade do cuidado^(5)^.

Os estudantes de enfermagem e enfermeiros evidenciam que a relação de cuidados, a proximidade e o envolvimento emocional atenuam o estresse, funcionando como mecanismo compensatório, pois não experienciam apenas o sofrimento, mas também momentos gratificantes no processo relacional, como a promoção da cura. Sendo assim, as emoções negativas e exaustão emocional, decorrentes da práxis da enfermagem, são atenuadas por emoções positivas como a gratificação e a satisfação^(6)^.

Nessa lógica, a Psicodinâmica do Trabalho (PDT) privilegia a clínica como modo de construção do conhecimento, de interpretação e de análise do ambiente laboral, colocando-se como instrumento capaz de compreender o processo de saúde-doença^(7)^. Para a PDT, o trabalho é entendido como categoria ontológica com potencial de ser fonte de prazer ou de sofrimento^(8)^.

O prazer pode manifestar-se como um sentimento benéfico relacionado à estabilidade psíquica do trabalhador, a partir do momento que supera as dificuldades impostas pelo trabalho, através de habilidades adquiridas e ações de resolutividade em seu meio profissional^(8-13)^. No entanto, o sofrimento é considerado uma experiência emocional desagradável associada a sentimentos como desvalia, impotência, insatisfação e estresse, diante das condições e organização do trabalho que, ligadas às características das pessoas, podem acarretar no adoecimento psíquico^(9)^.

No contexto laboral da unidade de internação pediátrica, o vínculo entre enfermeiros com as crianças em processo de institucionalização, muitas vezes, é sobreposto e entrelaçado como uma relação “quase parental”^(14)^. Dessa maneira, é comum que essa relação entre trabalhador e criança gere satisfação, mas também sofrimento, devido ao aumento da carga psíquica advinda do laço afetivo com essas crianças que estão internadas por um longo período de tempo sem acompanhantes.

Foram encontrados estudos com as seguintes temáticas: criança em instituição de acolhimento na perspectiva da equipe multidisciplinar^(2)^; criança hospitalizada dependente de tecnologia na visão de familiares e equipe de enfermagem^(3)^; o sentimento do enfermeiro no cuidado à criança hospitalizada sem acompanhante em estudo de revisão integrativa^(4)^; o papel da enfermagem em instituição de acolhimento para crianças na ótica da equipe multidisciplinar^(5)^. Além disso, estudos acerca da temática da PDT envolvendo prazer e sofrimento em unidade oncopediátrica^(15)^, em unidade de terapia pediátrica^(16)^ e em serviço de emergência pediátrica^(17)^ foram comuns nas buscas, por serem campos muito explorados na literatura. No entanto, estudos que abordem a temática envolvendo o cuidado de enfermeiros a crianças hospitalizadas que estão em processo de institucionalização utilizando o referencial da PDT não foram encontrados na literatura, justificando a relevância deste estudo.

Diante disso, entende-se a importância de realizar pesquisas que investiguem, sob a ótica da PDT, a repercussão que o cuidado ofertado pela equipe de enfermagem a crianças em processo de institucionalização tem na vida desses trabalhadores. A partir dessas considerações, o presente estudo buscará responder à seguinte questão norteadora: quais os fatores de prazer e sofrimento do enfermeiro no cuidado à criança hospitalizada em processo de institucionalização em uma unidade pediátrica?

Pretende-se que os resultados deste estudo auxiliem no conhecimento das especificidades diante dos sentimentos de prazer e sofrimento desses trabalhadores à luz da PDT. O estudo pode contribuir, ainda, para a discussão sobre questões relacionadas à saúde do trabalhador do local estudado, na medida em que dá visibilidade ao sofrimento dessa categoria, contribuindo para o desencadeamento de um processo de reflexão sobre o tema.

## OBJETIVOS

Analisar os fatores de prazer e sofrimento do enfermeiro no cuidado à criança hospitalizada em processo de institucionalização em unidade pediátrica.

## MÉTODOS

### Aspectos éticos

O estudo foi aprovado pelo Comitê de Ética em Pesquisa (CEP) da instituição de saúde, respeitando os itens que constam na Resolução nº 466/12^(18)^ e na Resolução nº 510/2016^(19)^ do Conselho Nacional de Saúde (CNS). Aos participantes, foi assegurada a preservação da identificação e fornecido o Termo de Consentimento Livre e Esclarecido (TCLE), com cópias de mesmo teor para o entrevistado e o pesquisador.

### Tipo de estudo

Estudo de cunho qualitativo do tipo descritivo. A pesquisa qualitativa preocupa-se com o que não pode ser quantificado, ou seja, trabalha com um universo de significados, o que corresponde às relações, processos e fenômenos que não podem ser reduzidos à operacionalização de variáveis. Ela visa explorar os significados das ações e relações humanas, lado que não é perceptível e captável em estatísticas^(20)^.

A pesquisa utilizou o guia *COnsolidated criteria for REporting Qualitative studies* (COREQ) para desenvolvimento e escrita do manuscrito. Assim, atendeu rigorosamente aos 32 critérios dos três domínios: equipe de pesquisa e reflexividade, conceito do estudo, análise e resultados que permitiram a qualificação do estudo^(21)^.

### Cenário do estudo

O estudo foi realizado em hospital universitário do Sul do Brasil, na unidade de internação pediátrica pertencente ao Serviço de Enfermagem Pediátrica. A unidade possui 28 leitos, composta por seis enfermarias com quatro leitos em cada uma, totalizando 24 leitos destinados exclusivamente ao Sistema Único de Saúde (SUS) e dois quartos com dois leitos semi-privativos, destinados aos convênios. Nesta unidade, ocorrem hospitalizações de crianças com doenças e comorbidades crônicas, inclusive doenças genéticas, cuidados clínicos agudos, prée pós-cirúrgicos.

Por ser considerada uma unidade semicrítica^(22)^, a instituição preconiza a permanência de um profissional da equipe de saúde, em especial o enfermeiro, em tempo integral, em cada uma das enfermarias. Dessa maneira, a unidade se tornou uma referência para crianças com histórico social de vulnerabilidade que, em alguns casos, estão desacompanhadas, e essa permanência do profissional naturalmente gera uma aproximação entre as crianças hospitalizadas e esses trabalhadores. Tal motivo influenciou a escolha do local.

### Participantes do estudo

A unidade possui 13 enfermeiros, e todos foram convidados para participar do estudo. Estabeleceram-se como critérios de inclusão os enfermeiros que estivessem exercendo funções de cuidado à criança hospitalizada em processo de institucionalização e que possuíssem contrato efetivo igual ou maior de seis meses de trabalho para maior familiarização com casos do interesse do estudo. Os critérios de exclusão foram enfermeiros que estivessem em licença saúde, licença gestação ou em período de férias.

A escolha dos entrevistados foi do tipo intencional por convite, envolvendo a seleção de participantes que compartilham características particulares e têm o potencial de fornecer dados ricos, relevantes e pertinentes à questão de pesquisa^(20)^. Cabe ressaltar que optou-se por entrevistar os enfermeiros da unidade devido ao fato de que esses profissionais assumem protagonismo no cuidado, uma vez que convivem com a criança à beira-leito em tempo integral.

Após a aplicação dos critérios de inclusão e exclusão, um enfermeiro encontrava-se em licença-saúde em decorrência de sintomas gripais, e outro enfermeiro havia sido recém-contratado, não havendo uma experiência de no mínimo seis meses. Coincidentemente, ambos enfermeiros excluídos devido aos critérios de seleção eram do sexo masculino. Sendo assim, 11 enfermeiras atenderam aos critérios de seleção e participaram da pesquisa.

### Coleta e organização dos dados

As entrevistas ocorreram após a apresentação do projeto de pesquisa a todos os enfermeiros da unidade em reunião de equipe. Dessa maneira, foram agendadas entrevistas individuais com os profissionais interessados a participar do estudo em um horário fora da jornada de trabalho.

A coleta das informações foi realizada nos meses de abril a maio de 2023, com entrevistas de duração entre 18 e 26 minutos, e foram aplicadas por uma das pesquisadoras com o auxílio de uma bolsista de iniciação científica, ambas com experiência prévia em coleta de pesquisa qualitativa. As entrevistas ocorreram de maneira presencial, em uma sala de reuniões da unidade, sem ruídos e com garantia de privacidade, sendo gravadas e transcritas na íntegra para melhor análise e leitura do material.

O instrumento de coleta de dados foi entrevista semiestruturada contendo questões fechadas sobre o perfil dos profissionais e questões abertas sobre os sentimentos e fatores de prazer e sofrimento despertados ao cuidar de uma criança hospitalizada em processo de institucionalização na unidade pediátrica.

As profissionais entrevistadas foram codificadas pela letra “T”, de trabalhador, seguido pelo número de acordo com a sequência que as entrevistas ocorreram (por exemplo, T1, T2, e assim sucessivamente).

### Análise dos dados

Os dados foram analisados de acordo com a análise de conteúdo temática, proposta por Minayo^(20)^, e o processo de análise se desdobrou em três etapas: pré-análise; exploração do material; e tratamento dos resultados obtidos e interpretação.

A primeira etapa consistiu na retomada das hipóteses e dos objetivos iniciais da pesquisa. Durante essa fase, foi realizada uma leitura flutuante para contato direto e intenso com o material de campo, a fim de compreender o conteúdo e a constituição de um *corpus*. Ainda na fase pré-analítica, determinaram-se as unidades de registro (palavra-chave), a unidade de contexto (a delimitação do contexto de compreensão da unidade de registro), os recortes, a forma de categorização, a modalidade de codificação e os conceitos teóricos mais gerais que orientaram a análise^(20)^.

A segunda etapa consistiu em agrupamento de categorias onde o conteúdo foi organizado, visando alcançar a compreensão do texto, separando em categorias responsáveis pela especificação dos temas^(20)^.

Por fim, na terceira etapa, evidenciaram-se as informações obtidas e, a partir delas, foram realizadas inferências e interpretações, relacionando-as em torno de novas dimensões teóricas baseadas em literatura^(20)^.

Após a análise de conteúdo temática, emergiram duas categorias analíticas: *Fatores geradores de prazer no trabalho ao cuidar de crianças hospitalizadas em processo de institucionalização*; e *Fatores geradores de sofrimento no trabalho ao cuidar de crianças hospitalizadas em processo de institucionalização*.

As análises e discussões dos resultados foram realizadas à luz da PDT, servindo como referencial teórico para o estudo. A PDT foi idealizada pelo médico psiquiatra francês Christophe Dejours, que estuda as relações dinâmicas entre o trabalho e os processos subjetivos e intersubjetivos que surgem da relação entre a organização psíquica das pessoas com o processo laboral^(8-13)^.

## RESULTADOS

A faixa etária das participantes variou entre 26 e 46 anos, e todas as participantes eram do sexo feminino. Quanto ao tempo de formação das trabalhadoras, uma enfermeira possuía três anos de formação; uma possuía quatro anos; três possuíam 13 anos; duas possuíam 18 anos; três possuíam 20 anos; e uma possuía 21 anos, sendo a enfermeira com maior tempo de formação na unidade do estudo no período da coleta de dados. Em relação ao tempo de trabalho na unidade pediátrica da instituição de saúde, uma enfermeira possuía seis meses de contrato de trabalho (sendo um dos requisitos para ser incluída no estudo), e a profissional com maior tempo de contrato possuía 16 anos na unidade estudada.

### Fatores geradores de prazer no trabalho ao cuidar de crianças hospitalizadas em processo de institucionalização

Diante do exposto e a partir das narrativas das enfermeiras da unidade de internação pediátrica estudada, emergiram as seguintes subcategorias: realização profissional; atuação na educação em saúde; e afeto e vínculo com a criança.

A realização profissional se dá por diversas razões, segundo as trabalhadoras entrevistadas. Um dos fatores geradores de prazer e contentamento é a sensação de dever cumprido e sair do ambiente de trabalho com o sentimento de que fez a diferença no cuidado da criança que se encontra hospitalizada.

*São crianças que precisam mais da gente. Me dá satisfação saber que aquele cuidado para aquela criança é muito importante* [...]. *Então, quando vou fazer o procedimento, vou aproveitar para conversar e até pegar no colo, se possível. Isso para a criança é importante, me dá satisfação.* (T5)

*Quando elas internam, estão bem debilitadas, com febre, com piora ventilatória. E conforme os dias vão passando, a gente vai fazendo os cuidados, vai tendo resultado e ficando feliz, porque essa criança chegou em um estado de saúde ruim e vai melhorando. A gente se sente feliz, porque era uma criança agitada, só chorava e dormia e daqui a pouco está interagindo contigo* [...]. *É muito gratificante, é um sentimento de alegria.* (T4)

As entrevistadas referem prazer ao demonstrarem a importância que o trabalho tem em suas vidas e ao verbalizarem o quanto gostam do que fazem. Sentem-se realizadas profissionalmente ao identificarem-se com o trabalho, obtendo prazer ao fazer diferença no processo de hospitalização da criança, sobretudo quando há a melhora do estado de saúde das crianças.

Ainda de acordo com as entrevistadas, visualizar o avanço clínico e o progresso da criança, que antes estava bem debilitada, é um aspecto que gera prazer e satisfação, pois os resultados favoráveis tiveram sua participação e investimento no cuidado. Dessa forma, é possível perceber que a participação ativa para a melhora do paciente é condição geradora de prazer e satisfação, pois os desfechos, na maioria das vezes, com impactos positivos e favoráveis, são motivadores para a continuidade do trabalho.

A atuação na educação em saúde e o treinamento foram destacados como uma das competências das trabalhadoras entrevistadas no preparo para alta hospitalar dessas crianças. Ao desenvolver esse trabalho, elas relatam que se sentem confortáveis, pois estão encaminhando a criança para cuidadores que serão capacitados para fazê-los na instituição que ela será acolhida.

*Uma coisa que conforta e que eu fico muito feliz é quando eu consigo conhecer o pessoal da instituição, quando eles vêm antes. A gente consegue até fazer um treinamento ou alguma coisa antes da saída da criança, eu acho que isso dá uma sensação de conforto* [...]*. Quando vem a enfermeira da instituição ou assistente social e fisioterapeuta, a gente consegue fazer a “passagem de plantão”. Isso é muito importante para mim, eu me sinto bem quando consigo. Parece que está completo teu trabalho. Tu devolveu para a sociedade, ou para instituição, mas tu passou o paciente. Isso é interessante, pois senão eu acho que parece meio incompleto ou duvidoso.* (T2)

As enfermeiras reconhecem a importância da relação interpessoal nas práticas em saúde para compreender a situação de saúde/doença da criança (muitas vezes dependentes de tecnologias em saúde) e realizar o treinamento dos cuidados. Assim, planejar o processo de alta hospitalar e instrumentalizar os futuros cuidadores da instituição que a criança for transferida proporciona a sensação de realizar um trabalho completo, gerando prazer no trabalho.

O afeto e o vínculo com a criança também foram apontados pelas entrevistadas como fatores que geram prazer na medida em que se doam para aquela criança que vive por longos períodos hospitalizada e desacompanhada. As enfermeiras verbalizaram que atitudes como pegar no colo e conversar olhando no olho são detalhes que aproximam e fazem a diferença no cuidado.

*Pela falta que eles têm de um carinho a mais, de ter o cuidado da família. Então, geralmente temos que fazer, além do cuidado de enfermagem, esse papel de maternagem. A gente acaba se tornando cuidador referência dessa criança. Então, acaba se apegando muito mais* [...]*. A gente acaba tentando suprir alguma questão que falta na vida dessa criança.* (T6)

*Eu me sinto muito mais na obrigação de oferecer o máximo de carinho, afeto e integralidade. A nossa rotina é muito corrida, mas eu consigo conciliar de uma forma que me traz um prazer muito grande. Eu gosto muito de poder me doar para essas crianças especiais* [...]. *O que a gente puder fornecer, eu acho válido, faz a diferença. O carinho que aquela criança não tem, pegar no colo, olhar no olho. Esses pequenos detalhes me trazem prazer, porque eu sei que, no momento que eu consigo minimizar o sofrimento de uma criança, isso é algo que me faz muito bem.* (T9)

É uma tarefa quase inevitável trabalhar em uma unidade pediátrica e não criar laços com a criança que está hospitalizada. O cenário corrobora ainda mais quando essa mesma criança se encontra desacompanhada e necessitando de uma atenção especial. Sendo assim, a relação formada por afeto, afinidade, interação, aproximação e vínculo gera sentimento de prazer às profissionais que encontram um momento em meio à rotina atarefada para realizar a maternagem. Além disso, a “maternagem” nada mais é do que um trabalho não prescrito contratualmente, mas que se torna real nas rotinas das trabalhadoras pediátricas, gerando prazer.

### Fatores geradores de sofrimento no trabalho ao cuidar de crianças hospitalizadas em processo de institucionalização

A partir das entrevistas com as enfermeiras da unidade do estudo, emergiram as seguintes subcategorias: casos de violência; desvinculação e desapego; e procedimentos invasivos.

As enfermeiras entrevistadas verbalizaram que trabalhar com casos que envolvem violência infantil é muito difícil e sofrido, visto que surgem, em muitos momentos, sentimentos de pena e revolta, além da insegurança relacionada à falta de conhecimento para o cuidado dessas crianças nesse contexto.

*Desperta sentimentos diferentes, quando uma criança passa por algum tipo de abuso, tanto psicológico quanto sexual, a gente fica revoltado, porque é uma pessoa que depende de um adulto e está sofrendo uma agressão. É um sentimento de revolta* [...]. *As crianças acabam verbalizando algumas coisas que tu não espera durante a brincadeira. A gente não foi capacitado para quem a gente tem que acionar para tentar resolver esses problemas* [...]. *Eu acho que a gente deveria ser instrumentalizado para saber o que fazer quando acontecem essas situações, porque é pouco trabalhado durante nossa formação como enfermeiro.* (T4)

*Criança que sofre violência é uma coisa que mexe muito comigo. É uma coisa que não consigo entender. Muitas vezes, chega com “suspeita de” e os pais estão juntos* [...]. *Existe um sentimento de repulsa, só de saber o que pode ter acontecido com a criança, mas eu não julgo, não falo nada para eles, mas sinto isso. Causa um bloqueio.* (T11)

É nítido, através dos depoimentos das trabalhadoras, que o atendimento e o cuidado aos casos de violência na infância em meio ao cotidiano laboral são cansativos e desgastantes. A alta sobrecarga emocional e psíquica dispendida é passível de sofrimento, capaz de induzir a um processo de adoecimento causado pelo ambiente de trabalho. As enfermeiras também reforçaram nas entrevistas que a falta de preparo e de experiência para o atendimento desses casos acaba interferindo no modo de cuidar, pois o desconhecimento da rede intra e extra-hospitalar gera sentimentos negativos e insegurança às trabalhadoras.

As entrevistadas destacaram que as experiências vividas no trabalho, muitas vezes, causam impotência, que advém de situações em que o profissional, por mais que preste a assistência qualificada, não consegue modificar o estado em que o paciente se encontra. Além disso, realizar o processo inverso, de desvinculação e desapego, no momento da institucionalização, é fator de sofrimento para o profissional que cuidou por um longo período da criança.

*É um desapego difícil* [...]. *Algumas institucionalizações são mais rápidas, e outras mais demoradas, e essas crianças que ficam muito tempo desacompanhadas, a gente acaba substituindo a família em vários momentos. É sofrida a separação.* (T2)

*Tristeza pelo afastamento, um receio. A gente sente o fato dela* (a criança) *estar saindo, porque a enfermagem, até então, estava fazendo o cuidado dessa criança, e agora ela vai sair daqui. Então, acho que a tristeza é um fator de sofrimento no processo de separação, institucionalização.* (T6)

A enfermagem sofre ao precisar reprimir seus sentimentos e trabalhar internamente o processo da alta hospitalar e a quebra do vínculo desenvolvido ao longo do cuidado com a criança, no momento em que a mesma é transferida para a instituição de acolhimento. Além disso, sentem insegurança pelo desconhecimento acerca do futuro da criança, aumentando a carga psíquica no trabalho.

Os procedimentos invasivos representam uma das principais experiências negativas no contexto hospitalar, e provocam ansiedade e medo nas crianças, gerando grande sofrimento na trabalhadora que precisa realizá-lo.

*Quando uma criança que já está muito tempo internada, muito manipulada, questionamos: “Será que eu precisava realmente coletar essa urina por cateterismo?”. Tu fica pensando que talvez possa trazer mais sofrimento para aquela criança ao invés de alívio, que a vida lá fora foi tão sofrida; em alguns casos, acho que isso atrapalha. Automatizamos esses processos para não sofrer tanto, mas não são coisas legais.* (T2)

*A gente fica pensando como essa criança ficou, se ela ficou bem. Tu fez um procedimento, tu sai do plantão naquele dia, viu que a criança está mais chorosa, com fácies de dor, tu fica preocupada* [...]. (T4)

O desafio das enfermeiras de cuidar de uma criança por meio de procedimentos dolorosos traz para a trabalhadora entrevistada ainda mais sofrimento, tendo em vista que a mesma sabe que a criança já padece em decorrência de questões prévias para além da hospitalização. Sendo assim, conforme o relato das profissionais, a automatização dos procedimentos é utilizada como uma forma de diminuir o seu próprio sofrimento.

## DISCUSSÃO

O prazer advindo da realização profissional das entrevistadas ocorre no momento em que conseguem acompanhar a melhora do estado de saúde da criança. Além disso, a troca de informações com a equipe da instituição que vai receber a criança também foi apontada como um aspecto de prazer, pois as trabalhadoras conseguem realizar a transição do cuidado através da educação em saúde do futuro cuidador no momento da alta da criança. Em relação ao tempo prolongado no ambiente hospitalar, essas profissionais também criam vínculo com essas crianças, uma vez que evidenciam a falta de acompanhante, fazendo com que as mesmas despendam mais tempo oferecendo carinho, afeto e integralidade no cuidado.

A satisfação profissional dos enfermeiros é condicionada por uma multiplicidade de fatores: é sentir-se bem com o que se faz, trabalhar feliz, realizado, fazer aquilo que gosta, ter as suas necessidades atendidas e ter as suas expectativas no trabalho contempladas. Assim, a satisfação ocorre quando os seus desejos profissionais vão ao encontro das metas e filosofia da instituição, quando atinge um resultado esperado ou quando o fato desejado não é descartado^(23)^.

O reconhecimento da qualidade do trabalho realizado é a resposta às expectativas subjetivas que os profissionais carregam. Assim, os desafios e o cansaço podem ser amenizados diante do sentimento de prazer com o reconhecimento no ambiente laboral. Dessa forma, trabalhar não é apenas produzir, mas é também transformar a si mesmo^(13)^, na busca de um objetivo em comum; neste caso, a melhora do estado de saúde da criança.

As sensações de satisfação e gratificação apresentam-se no momento em que o profissional compreende o restabelecimento da saúde do paciente como consequência das intervenções de enfermagem durante o período de hospitalização. Considera-se que o cuidado não ocorre de maneira isolada, sendo um processo interativo, exigindo disponibilidade, confiança e receptividade por parte do trabalhador^(15)^.

Quando chega o momento da institucionalização, a criança precisa se adaptar a uma nova realidade, e os cuidadores possuem um papel fundamental na compreensão das particularidades e potencialidades de cada acolhido. Complementarmente, a instituição de destino tem a função de propiciar cuidados e educação, fornecendo recursos de enfrentamento às dificuldades e favorecendo o desenvolvimento afetivo, cognitivo e social dessas crianças^(24)^.

Dessa maneira, para o estabelecimento de uma rede coordenada, é essencial uma ação comunicativa, implicando relações interpessoais de interdependência, as quais garantem o acesso e a continuidade do cuidado e, principalmente, evitam procedimentos desnecessários ou a sua duplicação^(25)^.

Contribuindo com isso, é indispensável que os profissionais de enfermagem realizem treinamentos para aqueles que irão receber a criança dependente de cuidados de saúde de um serviço hospitalar, com conhecimentos técnicos específicos que possam respaldar os cuidadores que prestarão esse cuidado^(24)^.

Desse modo, a satisfação por executar uma tarefa prescrita, incluindo ações do trabalho real, corrobora para a qualidade nas relações interpessoais, a partir da organização do trabalho, e repercute em vivências positivas pelos indivíduos em seu exercício profissional^(8)^. Assim, o prazer é extraído do sucesso do trabalho, podendo ser resultado das boas relações nos contextos laborais, ou seja, do reconhecimento do seu trabalho ao realizar educação em saúde^(17)^.

Quando somada a essa experiência o fato de a criança possuir uma rede de apoio frágil ou até mesmo não possuir acompanhante nesse período, torna-se um desafio ainda maior para os trabalhadores que cuidam dela. Nesse sentido, o profissional de saúde, ao estabelecer vínculo com a criança, pode traçar possibilidades que permitam manter o desenvolvimento infantil em todos os seus aspectos. Assim, enfermeiros estabelecem uma relação interpessoal com os futuros cuidadores, bem como com as crianças hospitalizadas, que por si só já experienciam situações que causam estresse e desestabilização emocional^(26)^.

Desse modo, o vínculo e o afeto, embora não estejam prescritos no fazer do profissional de enfermagem, ocorrem no cotidiano de trabalho, uma vez que o enfermeiro permanece boa parte da sua jornada à beira leito junto com a criança. Além disso, a criatividade é essencial para transformar o trabalho prescrito em trabalho real, pois, sem essa capacidade, o ambiente laboral pode se tornar limitante e fatigante^(12)^.

Estabelecer vínculo e envolvimento emocional torna o trabalho dos enfermeiros no dia a dia mais eficaz, uma vez que possibilita maior confiança na relação entre a criança e o profissional. O vínculo em si é uma via dupla, ao passo que aproxima e qualifica o cuidado, e também potencializa os recursos humanos utilizados pelos trabalhadores, ultrapassando os protocolos gerais e levando a um maior empenho no cuidado^(23)^.

Considerando o fato de que todas as entrevistadas eram do sexo feminino, aquelas que também eram mães pontuaram aspectos comuns em suas falas, ou seja, uma breve associação do cuidado desempenhado no setor hospitalar com a maternagem, tanto no modo de agir quanto no envolvimento afetivo com a criança. Historicamente, o vínculo criado entre profissionais pediátricos e as crianças é mais intenso, quando comparado a outras áreas, fazendo com que essas trabalhadoras se lembrem dos filhos que estão em casa. Nesse sentido, o cuidado dispensado à criança exige dessas enfermeiras o desenvolvimento de habilidades interativas que contribuem diretamente para o vínculo e conquista da confiança, e isso pode implicar intensidade dos sentimentos gerados, devido ao tempo de cuidado prolongado^(27)^.

Assim, a mobilização subjetiva é o processo por meio do qual o trabalhador se engaja no trabalho, lança mão de sua subjetividade, da sua inteligência prática e do coletivo de trabalho para transformar as situações causadoras de sofrimento (a carência de carinho e da família de origem da criança) em situações geradoras de prazer (vínculo e reciprocidade de afeto entre trabalhador e criança)^(7)^. Sendo assim, as situações que ocasionam sofrimento aos trabalhadores acontecem quando há falha na intermediação entre as expectativas do trabalhador e a realidade imposta pela organização e gerência do trabalho^(16)^.

A identificação subjetiva com o trabalho pressupõe que a atividade tenha sentido gratificante para o sujeito na forma de retribuição simbólica que, ao fazer uso da sua personalidade e inteligência, confronta a realidade subjetiva do trabalho e encontra formas de transformar o seu sofrimento em algo que seja positivo, denominando-se sofrimento criativo. Já o sofrimento patogênico é desenvolvido a partir do momento em que o trabalhador não possui liberdade para atuar e transformar sua realidade laboral, utilizando estratégias defensivas para conseguir se adaptar ao trabalho, as quais são alienantes ao trabalhador^(8)^.

O sofrimento foi evidenciado neste estudo a partir de experiências vivenciadas pelas trabalhadoras ao lidar com casos de violências cometidos, das mais variadas formas, com as crianças. Ademais, por possuírem forte vínculo com as crianças, sentimentos negativos surgem no rompimento desta conexão, no momento da separação para institucionalização, gerando um desapego forçado e sentimento de despertencimento. Além disso, ao realizar procedimentos invasivos, essas profissionais se abalam ao gerar mais desconforto para essa criança já fragilizada, muitas vezes automatizando esse processo para aliviar sua carga psíquica.

O contato com a violência contra a criança desperta no enfermeiro sentimento de sofrimento, uma vez que ele busca entender a situação em que ela ocorre. Dessa maneira, buscar compreender esse contexto é, para o profissional, uma complexa tarefa, uma vez que a família deveria ser o alicerce, protegendo, acolhendo e proporcionando segurança e conforto ao longo do seu crescimento e desenvolvimento^(28)^.

Nesse cenário, quando o agressor é um membro da família, a abordagem à criança vítima de violência que está internada é de difícil manejo, visto que essas práticas se perpetuam por diferentes crenças e gerações. A tentativa de manter-se imparcial, sem julgar a família e seguir no cuidado da criança, gera sofrimento e desgaste emocional no trabalhador, causando aumento da carga psíquica e tornando o trabalho fatigante^(11)^.

Dessa maneira, lidar com questões de violência na infância é de grande impacto no ambiente laboral para o trabalhador, visto que a falta de autonomia e manejo é elemento que influencia negativamente a relação do enfermeiro para com o familiar, pois ainda há barreiras de formação e capacitação diante das situações de violência infanto-juvenil^(17)^.

De forma geral, os profissionais de saúde apresentam dificuldades em trabalhar com situações que envolvam violência, independentemente do seu nível de formação, e isso se dá pelo fato de a formação em saúde assumir uma visão biomédica, que atua na lógica curativa, não contemplando a complexidade do tema, a qual não é passível de medicalização, exigindo outras estratégias dos enfermeiros. Assim, a focalização de intervenções profissionais na tomada de decisão ao acolhimento institucional de crianças pode estar vinculada à dificuldade de manejo em situações que envolvem maior complexidade^(29)^, pois, muitas vezes, a “rede de suporte” da criança era a mesma que praticava a violência contra ela.

Assim, o trabalhador está sujeito a um desgaste emocional, tendo em vista as vivências que resultam da polivalência de cuidados, pois o trabalhador se vincula à criança durante todo o período de hospitalização^(30)^. Dessa forma, o papel desempenhado pelos trabalhadores com essas crianças é comparado com o de seus próprios filhos, com as mesmas preocupações e dedicação. No entanto, a vinculação afetiva com a criança que será acolhida possui como consequência a quebra do vínculo no momento da alta hospitalar a caminho da instituição de acolhimento. Esse processo causa desgaste psíquico, potencializando sentimentos de abandono e desmotivação para o profissional que precisa acostumar-se com a ausência da criança^(24)^.

Esses sentimentos podem ser vistos como sinônimo de limitação e impotência profissional no ambiente de trabalho. Os profissionais sentem-se frustrados e, além de lidarem com a experiência de separação da criança, lidam também com o inesperado e o trágico sentimento de despertencimento da situação que fazia parte da sua rotina laboral^(16)^.

Essa sensação de impotência vai além do ambiente hospitalar, pois o enfermeiro gostaria de poder contribuir com essas crianças além desse âmbito, acreditando que, para que a criança pudesse vivenciar uma vida melhor, seria necessário o apoio de outras redes de suporte. Assim, o trabalhador gostaria de ter autonomia de poder continuar a realizar seus cuidados para ela, mas seu papel como profissional implica determinadas atitudes que permanecem aquém de seus desejos^(28)^.

Apesar de os cuidados ligados ao corpo e à doença objetivarem a recuperação e a qualidade de vida das crianças hospitalizadas, os enfermeiros, em sua prática assistencial, podem realizar procedimentos invasivos que são necessários e fazem parte do tratamento, entretanto geram desconforto na criança e, consequentemente, podem gerar sofrimento ao próprio trabalhador^(31)^.

A criança em condições crônicas complexas acaba precisando ser puncionada (punções venosas e arteriais, verificação de glicemia), aspirada, mudada de posição, submetida à inserção de sondas, curativos, cateterismos, entre outros. Dessa maneira, a interface da técnica com a intersubjetividade é estratégica nesse ambiente complexo, no qual cuidar às vezes remete à dor, à invasão e à contenção^(32)^, podendo ativar reações de estresse e ansiedade tanto na criança quanto no enfermeiro, que precisa saber manejar a situação^(33)^. Sendo assim, imposições de domínio técnico e científico que se colocam na vivência do trabalho dessas profissionais, em um momento de grande fragilidade das crianças, podem fazer com que as trabalhadoras adoeçam psiquicamente.

Além disso, mesmo que os enfermeiros possuam considerável experiência na área, seguem tendo empatia pelo sofrimento dessas crianças como se fossem seus próprios familiares^(15)^. Assim, o trabalhador precisa lidar com a realidade e com as dificuldades impostas, reconhecendo o exercício livre de sua inteligência e habilidades para resolução de problemas^(10)^. É nesse momento que o sofrimento acomete o profissional, pois o vínculo criado desencadeia o sentimento de causar dor na criança.

Dessa maneira, ir até o leito para realizar apenas o procedimento não parece ser uma opção na pediatria (trabalho prescrito). É necessário ter uma relação de confiança, vínculo, proximidade ao tentar convencer a criança e auxiliar no processo de resiliência (trabalho real). Dessa forma, é na distância entre as normas e regras previstas e o modo de trabalhar na realidade que a possibilidade de sofrimento acontece^(11)^, pois o cuidado dessas crianças não se trata apenas do fazer técnico.

### Limitações do estudo

É importante ressaltar que o presente estudo apresenta algumas limitações, como a escolha de apenas uma determinada unidade de internação pediátrica de somente um hospital, pois pode ser que, em outras unidades que recebem crianças com o perfil do estudo, os trabalhadores tenham diferentes percepções de fatores de prazer e sofrimento. Além disso, a particularidade deste estudo ao entrevistar enfermeiras apresentou um recorte de apenas uma categoria profissional. Sendo assim, a inclusão de outros profissionais da equipe multidisciplinar, certamente, poderia agregar outras informações a respeito da PDT nessas unidades.

### Contribuições para as áreas da enfermagem, saúde ou políticas públicas

O estudo contribuiu para a discussão sobre questões relacionadas à saúde do trabalhador do local estudado, na medida em que dá visibilidade ao sofrimento dessa categoria. Além disso, promove a tentativa de auxiliar e proporcionar aos gestores da instituição subsídios e ferramentas para articulação e implementação de práticas que proporcionem reflexão sobre o tema no ambiente laboral, de acordo com as necessidades dos trabalhadores que se encontram em cenários desencadeantes de adoecimento devido à alta carga psíquica.

## CONSIDERAÇÕES FINAIS

As enfermeiras encontraram prazer na realização profissional, atuação na educação em saúde e afeto e vínculo com a criança. Esses aspectos reverberam sentimentos de prazer na trabalhadora a partir do momento que conseguem perceber que a atuação para a melhora do estado de saúde foi fruto de um trabalho contínuo, composto por vinculação e proximidade com a criança, gerando satisfação profissional. Além disso, a sensação de um trabalho completo e de missão cumprida ocorre quando as trabalhadoras realizam o treinamento dos futuros cuidadores da criança na instituição.

Já os casos de violência, desvinculação e desapego e procedimentos invasivos foram relatados pelas trabalhadoras como aspectos que geram sofrimento no cotidiano laboral. É explícito o sofrimento das profissionais quando atuam em casos de violência, e é uma tarefa muito difícil o processo de desapegar-se da criança quando ela é institucionalizada. Além disso, realizar procedimentos invasivos em uma criança que já sofreu fora dos muros hospitalares gera aumento da carga psíquica.

Considerando o cuidado na atenção hospitalar, é relevante o incentivo de novos estudos que promovam a implicação do cuidado a crianças em processo de institucionalização nos demais profissionais da equipe multidisciplinar, garantindo uma compreensão ainda mais acurada das repercussões desses sentimentos na saúde e processos de trabalho.

Portanto, a realização deste estudo oportunizou conhecer as especificidades diante dos sentimentos de prazer e sofrimento verbalizados pelas enfermeiras no cuidado de crianças hospitalizadas que estão em processo de institucionalização em unidade pediátrica de um hospital geral à luz da PDT.
